# Left Ventricular Hypertrophy in Aortic Stenosis: Early Cell and Matrix Regression 2 Months Post-Aortic Valve Replacement

**DOI:** 10.1161/CIRCIMAGING.124.017425

**Published:** 2024-12-04

**Authors:** Jonathan Bennett, George D. Thornton, Christian Nitsche, Francisco F. Gama, Nikoo Aziminia, Uzma Gul, Abhishek Shetye, Peter Kellman, Rhodri H. Davies, James C. Moon, Thomas A. Treibel

**Affiliations:** Institute of Cardiovascular Science, University College London, United Kingdom (J.B., G.D.T., C.N., N.A., R.H.D., J.C.M., T.A.T.).; Cardiovascular Imaging Department, Barts Heart Centre, London, United Kingdom (J.B., G.D.T., C.N., F.F.G., N.A., U.G., A.S., R.H.D., J.C.M., T.A.T.).; Division of Cardiology, Medical University of Vienna, Austria (C.N.).; National Heart, Lung, and Blood Institute, National Institutes of Health, Bethesda, MD (P.K.).; 1 Institute of Cardiovascular Science, University College London, London, United Kingdom; 2 Cardiovascular Imaging Department, Barts Heart Centre, London, United Kingdom; 3 Division of Cardiology, Medical University of Vienna, Vienna, Austria; 4 National Heart, Lung, and Blood Institute, National Institutes of Health, Bethesda, MD; 5 Department of Cardiothoracic Surgery, Barts Heart Centre, London, United Kingdom; 6 William Harvey Research Institute, Queen Mary University, London, United Kingdom

**Keywords:** aortic valve, aortic valve stenosis, hypertrophy, left ventricular, magnetic resonance imaging, myocardium

## Abstract

**BACKGROUND:**

In aortic stenosis, the myocardium responds with left ventricular hypertrophy, which is characterized by increased left ventricular mass due to cellular hypertrophy and extracellular matrix expansion. Following aortic valve replacement (AVR), left ventricular hypertrophy regression occurs, but early cellular and extracellular dynamics are unknown.

**METHODS:**

Patients with severe symptomatic aortic stenosis undergoing surgical or transcatheter AVR were prospectively recruited. Pre- and early post-AVR cardiac magnetic resonance imaging assessed left ventricular remodeling, global longitudinal strain, and T1 mapping to determine extracellular volume fraction and volume of cellular and extracellular compartments.

**RESULTS:**

In all, 39 patients (aged 71.4±9.8 years, male 79%, aortic valve peak velocity 4.4±0.5 m/s) underwent cardiac magnetic resonance before and at median 7.7 weeks post-AVR. Left ventricular mass index reduced significantly by 15.4% (*P*<0.001*), primarily driven by cellular compartment regression (18.7%, *P*<0.001*), with a smaller reduction in the extracellular compartment (7.2%, *P*<0.001*). This unbalanced regression led to an apparent increase in extracellular volume fraction (27.4±3.1% to 30.2±2.8%; *P*<0.001*). Although there was no significant change in global longitudinal strain post-AVR, an increase in extracellular volume fraction was associated with worsening of global longitudinal strain (Pearson r=0.41, *P*=0.01). Mode of intervention (transcatheter versus surgical) did not influence the above myocardial parameters post-AVR (all *P*>0.05). The asterisk in *P* values indicates a statistical significance of <0.05.

**CONCLUSIONS:**

Within 8 weeks of AVR for aortic stenosis, substantial left ventricular hypertrophy regression occurs involving both cellular and extracellular compartments, demonstrating the early myocardial adaptability to afterload relief. Cellular compartment regression is greater than extracellular regression, leading to an apparent increase in extracellular volume fraction. Mode of intervention did not affect degree of reverse remodeling, indicating that both are effective at resulting beneficial changes post-AVR.

**REGISTRATION:**

URL: https://www.isrctn.com; Unique identifier: NCT04627987.

**CLINICAL PERSPECTIVE**

Left ventricular hypertrophy, characterized by increased left ventricular mass, develops in severe aortic stenosis as a compensatory response to the increased pressure afterload from the progressively stenotic aortic valve. Left ventricular hypertrophy in aortic stenosis involves both cardiomyocyte hypertrophy and expansion of the extracellular matrix. Following aortic valve replacement (AVR), left ventricular mass regression occurs, though the early dynamics of cellular and extracellular changes remain unclear. In this study, we demonstrate that significant regression of left ventricular hypertrophy occurs within 8 weeks post-AVR, involving both cellular and extracellular compartments. Cellular regression outpaces that of the extracellular matrix, resulting in a relative increase in extracellular volume percentage. The mode of intervention (surgical or transcatheter) did not influence the degree of reverse remodeling, indicating both approaches lead to beneficial myocardial changes post-AVR. Our findings reveal that cellular hypertrophy is highly adaptable and regresses early after AVR. This highlights the potential benefit of targeting fibrosis in the early post-AVR period, as the unbalanced regression, marked by increased extracellular volume percentage, contributes to worsening global longitudinal strain in some patients. Early pharmacotherapeutic targeting of fibrosis could promote balanced regression in both compartments, leading to faster global longitudinal strain improvement and better long-term outcomes.

**See Editorial by Wong**

Left ventricular hypertrophy (LVH), defined by increased left ventricular mass (LVM), occurs in severe aortic stenosis (AS) to compensate for increased pressure afterload from the progressively stenotic aortic valve (AV). LVH in AS consists of both cardiomyocyte hypertrophy and expansion of the supporting extracellular matrix.^1^ The expanded extracellular matrix in AS comprises in part of replacement fibrosis, which occurs in response to cardiomyocyte loss alongside diffuse reactive fibrosis that occurs secondary to pressure or volume overload, and remodeling in areas remote from replacement fibrosis areas.^2,3^ Fibrosis, both replacement and diffuse reactive, is a potential arrhythmic substrate and contributes to ventricular wall stiffening and impaired oxygen and nutrient delivery that can contribute to functional impairment, heart failure, and increased risk of future cardiovascular events.

Following aortic valve replacement (AVR, surgical, or transcatheter), LVH regresses 15% to 20% at 1 year,^2,4,5^ and those with greater early LVM regression have half the rate of hospitalization post-AVR in the first year.^6^ Cardiovascular magnetic resonance (CMR) is an established tool that can quantify by T1 mapping the cardiac cellular compartment (myocytes, fibroblasts, and red blood cells) and extracellular compartment (extracellular matrix and blood plasma) by calculating the extracellular volume fraction (ECV%). A prior CMR study demonstrated that this LVH regression at 1 year is a combination of both cellular compartment regression and extracellular compartment regression, with a higher degree of cellular compartment regression relative to extracellular compartment regression.^2^

Considering that up to 50% of the LVM regression that is detected at 1 year has been reported to occur in the first 30 days,^6^ our study aims to determine the respective contributions of the cellular and extracellular compartments to this regression early after AVR.

## Methods

The data, analytical methods, and study materials will be made available to other researchers for purposes of reproducing the results or replicating the procedure. The data are available from the corresponding author upon reasonable request.

### Study Population

Adult (>18 years) patients with severe symptomatic AS were prospectively recruited at a single tertiary cardiac center (Barts Heart Centre, London, United Kingdom) between March 2021 and August 2024. The inclusion criteria were as follows: (1) at least one of the following echocardiographic features of severe AS (aortic valve area <1.0cm^2^, indexed aortic valve area=0.6 cm^2^/m^2^, peak velocity >4.0 m/s, or mean gradient >40 mm Hg); and (2) referred for surgical or transcatheter aortic valve replacement (SAVR/TAVR). The exclusion criteria were as follows: (1) more than moderate alternative valve disease than AS, (2) previous valve surgery/intervention, (3) diagnosis of cardiomyopathy (ie, hypertrophic cardiomyopathy and cardiac amyloidosis), (4) pregnancy/breastfeeding, and (5) CMR incompatible devices.

The study was approved by the UK National Research Ethics Service (19/LO/1849) and registered with ClinicalTrials.gov (NCT04627987). The study conformed to the principles of the Declaration of Helsinki, and all patients gave written informed consent pre-AVR.

### Patient Assessment

All patients underwent comprehensive pre-AVR clinical assessment, including clinical history, transthoracic echocardiography, and CMR. Patients were reassessed early (6–8 weeks) post-AVR with repeat CMR.

### Echocardiography

Echocardiography was used to assess AV severity and post-AVR prosthetic function following guidelines from the British Society of Echocardiography.^7^

### Cardiovascular Magnetic Resonance

**Figure 1.**
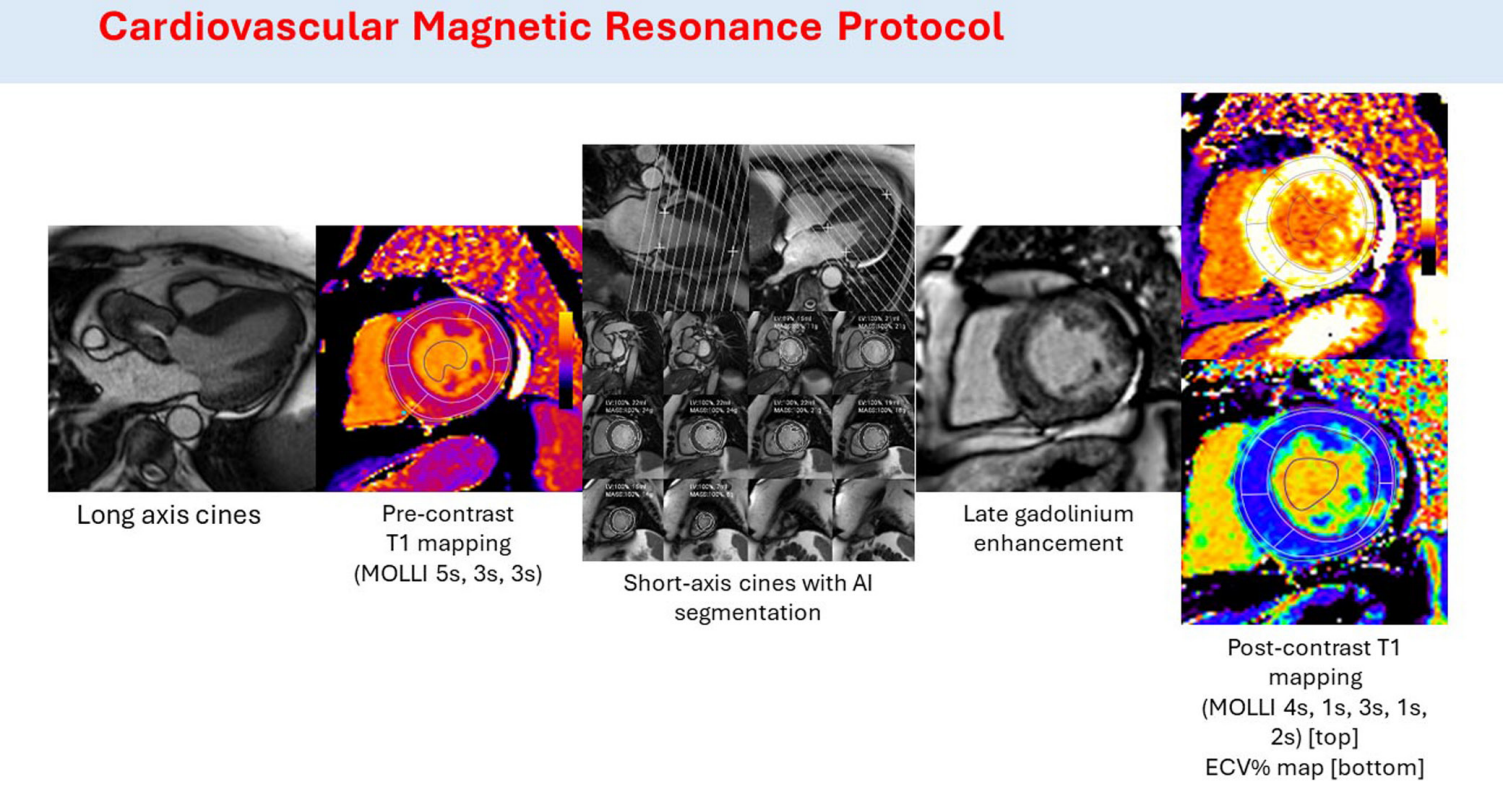
**Graphical representation of cardiovascular magnetic resonance protocol.** AI indicates artificial intelligence; ECV%, extracellular volume fraction; and MOLLI, modified look-locker inversion.

CMR was performed at 1.5T (Aera, Siemens Healthcare, Erlangen, Germany) by using a standard clinical scan protocol (Figure 1) comprising of long- and short-axis cine imaging, T1 mapping by Modified Look-Locker Inversion recovery before and after (15 minutes) a bolus of Gadolinium contrast (0.1 mmol/kg of gadoterate meglumine [gadolinium-DOTA, marketed as Dotarem, Guerbet S.A., Paris, France]), and late gadolinium enhancement (LGE) imaging performed 10 minutes after the contrast agent bolus.

### Imaging Analysis

CMR image analysis was performed using CVI42 software (version 5.12.1; Circle Cardiovascular Imaging, Calgary, Alberta, Canada). Assessment of left ventricular (LV) structure and function was undertaken using validated fully automated machine learning algorithms^8,9^ with inclusion of the trabeculations and papillary muscles within the LV mass. Left atrial volume index (LAVi) was manually contoured using the area-length method in the 4 and 2 chamber long-axis views.

Myocardial strain was performed on long-axis (4, 3, and 2 chamber) cine images using CVI42. The software automatically identified the LV endo- and epicardial contours, with manual corrections as needed. Global longitudinal strain (GLS) was derived from this automatic tracking over the cardiac cycle.

For T1 mapping, 3 short-axis T1 maps (base, mid, and apex) were manually contoured at the endocardial and epicardial borders with 10% offset to minimize partial voluming. The superior and inferior RV insertion points were identified for segmentation into the American Heart Association 16-segment model. Segments with infarct pattern LGE were excluded from global T1 calculation. The ECV% was determined using synthetic hemocrit as blood sampling was not consistently available at early follow-up due to COVID-19 restrictions.^10^ Extracellular volume index (ECVi) and cellular volume index (CellVi) were derived from indexed (to body surface area) LV mass (LVMi) divided by the specific gravity of myocardium (1.05 g/mL) then multiplied by ECV% or 1-ECV%, respectively.^2^

The presence and pattern (infarct pattern and noninfarct pattern) of LGE were qualitatively adjudicated by 2 experienced CMR clinicians (J.B., T.T.). Right ventricular insertion point LGE was not included in noninfarct pattern LGE adjudication.

### Statistical Analysis

Statistical analyses were performed using STATA, version 18.0 (STATAcorp, TX). Continuous variables were expressed as mean±SD with no substantial departures from normality found among the presented variables. Normality was assessed using Shapiro-Wilk. Categorical variables are expressed as percentages. Independent groups were compared using an independent *t* test. χ^2^ or Fisher exact test was used for categorical variables.

Pre- and post-AVR paired data were compared using paired *t* test for continuous variables or Wilcoxon signed-rank test for ordinal variables. Correlations were assessed using Pearson correlation coefficient (r) to evaluate the strength and direction of linear relationships between variables. Multivariable regression was performed using a forward selection approach to identify independent predictors. Candidate variables for inclusion were selected based on clinical relevance and those showing a borderline association (*P*<0.1) from the univariable regression analysis. Variables were added sequentially, and the final model included only those predictors that remained significant at a conventional threshold (*P*<0.05).

ANCOVA was used to assess whether the mode of intervention had a significant effect on follow-up variables, while adjusting for baseline variables. A 2-sided *P* value of <0.05 was considered significant.

## Results

### Study Population

A total of 41 patients with severe symptoms were recruited 2 patients were excluded from analysis due to their inability to complete follow-up CMR.

### Baseline Findings

Baseline demographic, clinical, and echocardiographic characteristics are described in Table 1. The mean age was 71.4±9.8 years with majority being male (31 [79.4%]). There was a mixed prevalence of comorbidities with 8 (20.5%) having a history of coronary artery disease, 8 (20.5%) having diabetes, and 26 (66.6%) having hypertension.

**Table 1.**
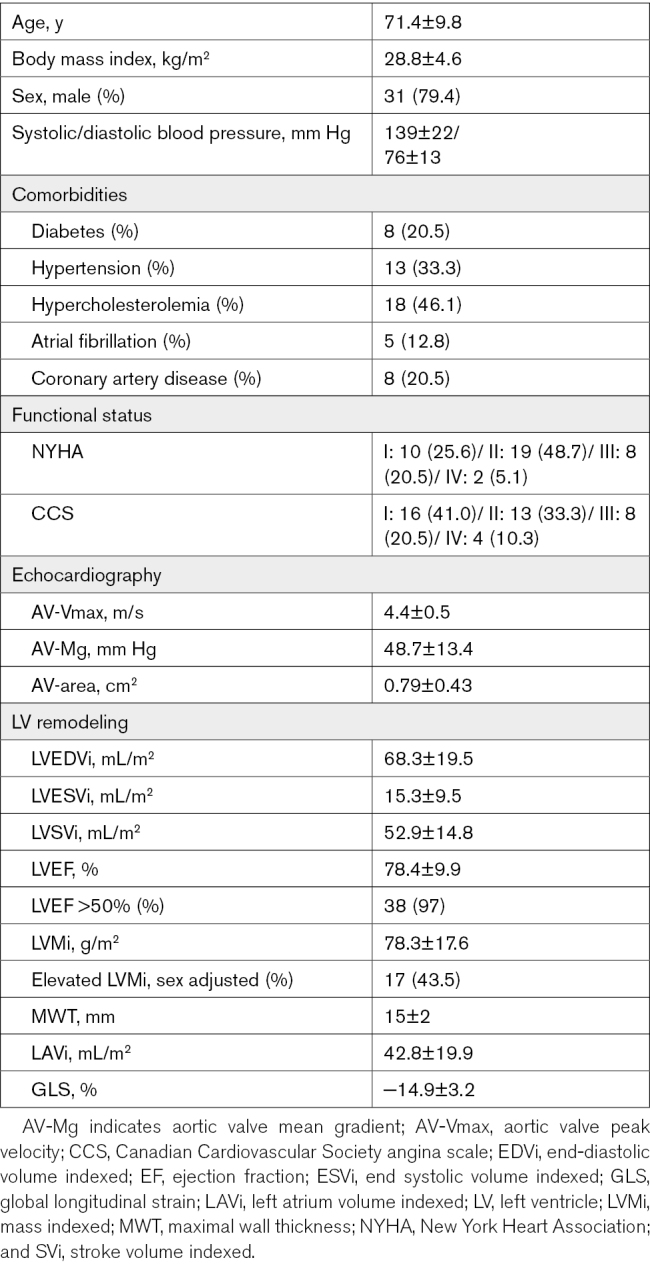
Baseline Characteristics

Patients were symptomatic with AS with the majority of the cohort being New York Heart Association class II or higher (29 [74.3%]) or Canadian Cardiovascular Society angina class II or higher (23 [58.9%]).

### Severity of AS

All patients had severe AS by echocardiography with mean peak aortic valve velocity (AV-Vmax) 4.3±0.5 m/s and mean aortic valve gradient (AV-Mg) 48.7±13.4 mm Hg.

### LV Remodeling at Baseline

The cohort exhibited a high proportion of elevated indexed LVM (17 [43.5%]) compared with sex adjusted reference ranges.^11^ The majority of our cohort had preserved left ventricular ejection fraction (LVEF) >50% (38 [97%]), and 1 (3%) patient had impaired LVEF with dilated LV end-diastolic volume index. Pre-AVR GLS was −14.9±3.2%, indicating impairment when compared with reference values in healthy subjects.^11,12^

Pre-AVR native T1 was 1037±37 ms and ECV% was 27.4±3.1%. Near 3 quarters of patients (29 [74.4%]) had noninfarct pattern LGE with a third (13 [33%)] having infarct pattern LGE.

### Intervention

Within our patient cohort, 23 (58.9%) underwent SAVR, while the remainder (16 [41.1%]) had TAVR. Coronary artery bypass was undertaken on 2 (5.1%) of the cohort. Patients undergoing TAVR were significantly older than SAVR patients (77.8±8.5 versus 66.9±8.1 years; *P*<0.001*) with increased LAVi (51.1±21.6 versus 35.8±15.6 mL/m^2^; *P*=0.021*) and systolic blood pressure (151±23 versus 130±16 mm Hg; *P*=0.002*). Before AVR, there was no significant difference between SAVR and TAVR in AV stenosis severity by echocardiography, LV remodeling, or tissue characterization (native T1, ECV%, CellVi, ECVi, and GLS; Table S1).

### Follow-Up Findings

Median follow-up was at 7.7 (6.7–9.2) weeks. At follow-up, patients reported functional status had significantly improved for both New York Heart Association and CCS (Wilcoxon sign-rank test *P*<0.001* for both; Table 2). There was significant reduction in AV-Vmax (4.4±0.5 to 2.4±9.4 m/s; *P*<0.001*) and AV-MG (48.7±13.4 to 12.3±6.1 mm Hg; *P*<0.001*). There was no significant change in systolic (139±22 to 136±23 mm Hg; *P*=0.58) or diastolic (76±13 to 74±11 mm Hg; *P*=0.16) blood pressure. TAVR patients had a mildly elevated AV-MG compared with SAVR after adjustment for baseline AV-MG (TAVR 14.5±4.9 versus SAVR 10.6±6.5 mm Hg; *P*=0.008; Table 3).

**Table 2.**
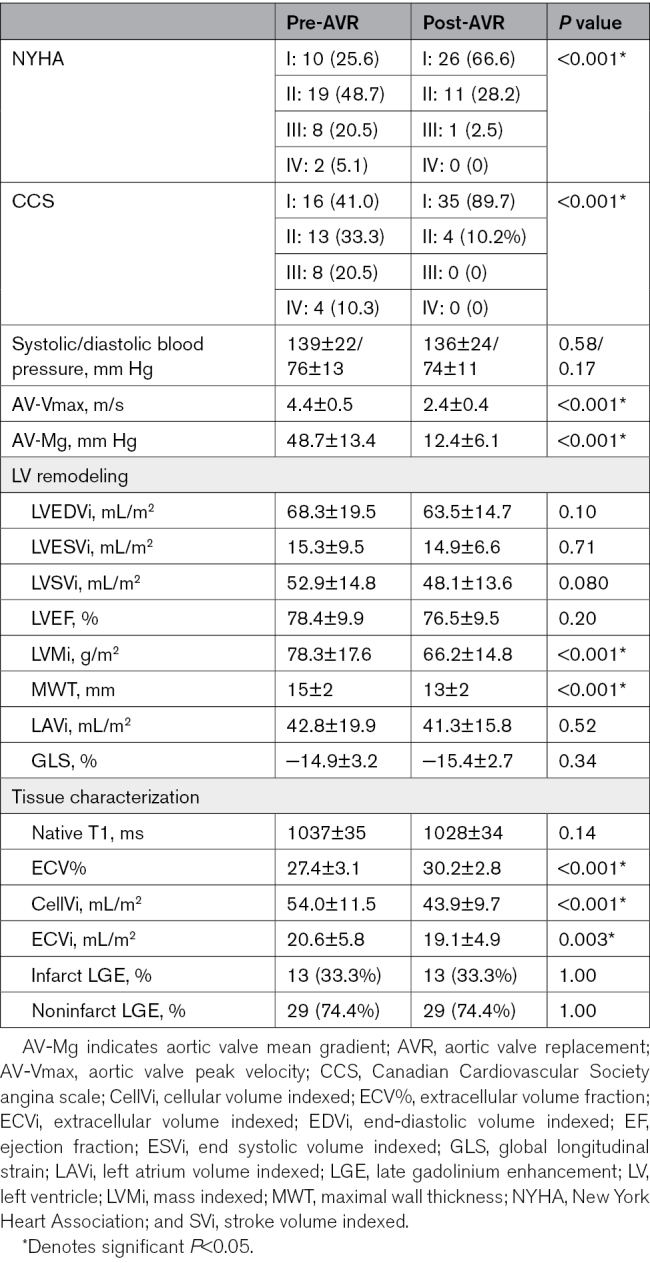
Changes After Aortic Valve Replacement

**Table 3.**
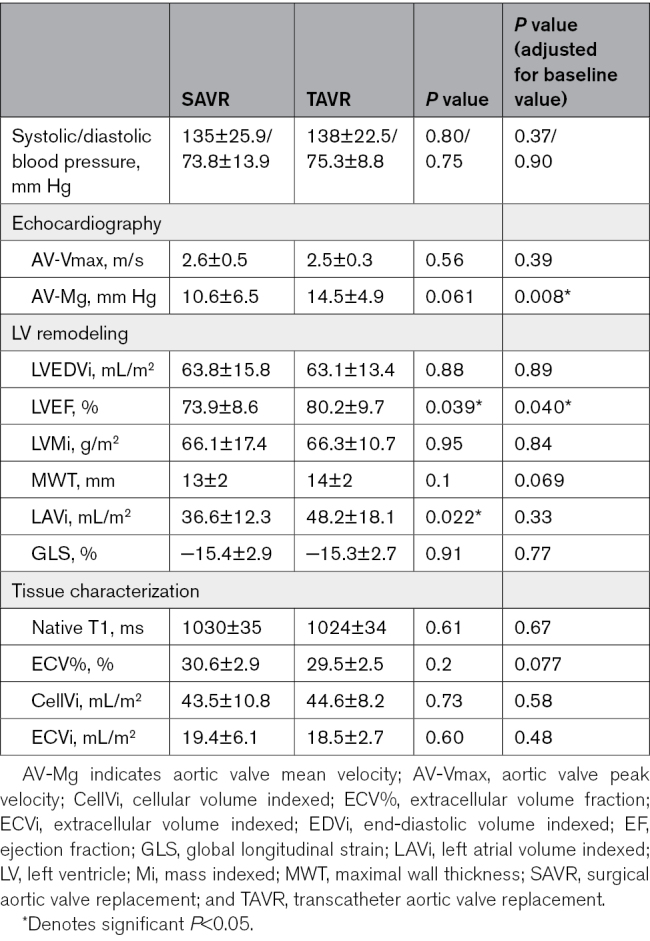
SAVR vs TAVR at Follow-Up

### LV Remodeling at Follow-Up

**Figure 2.**
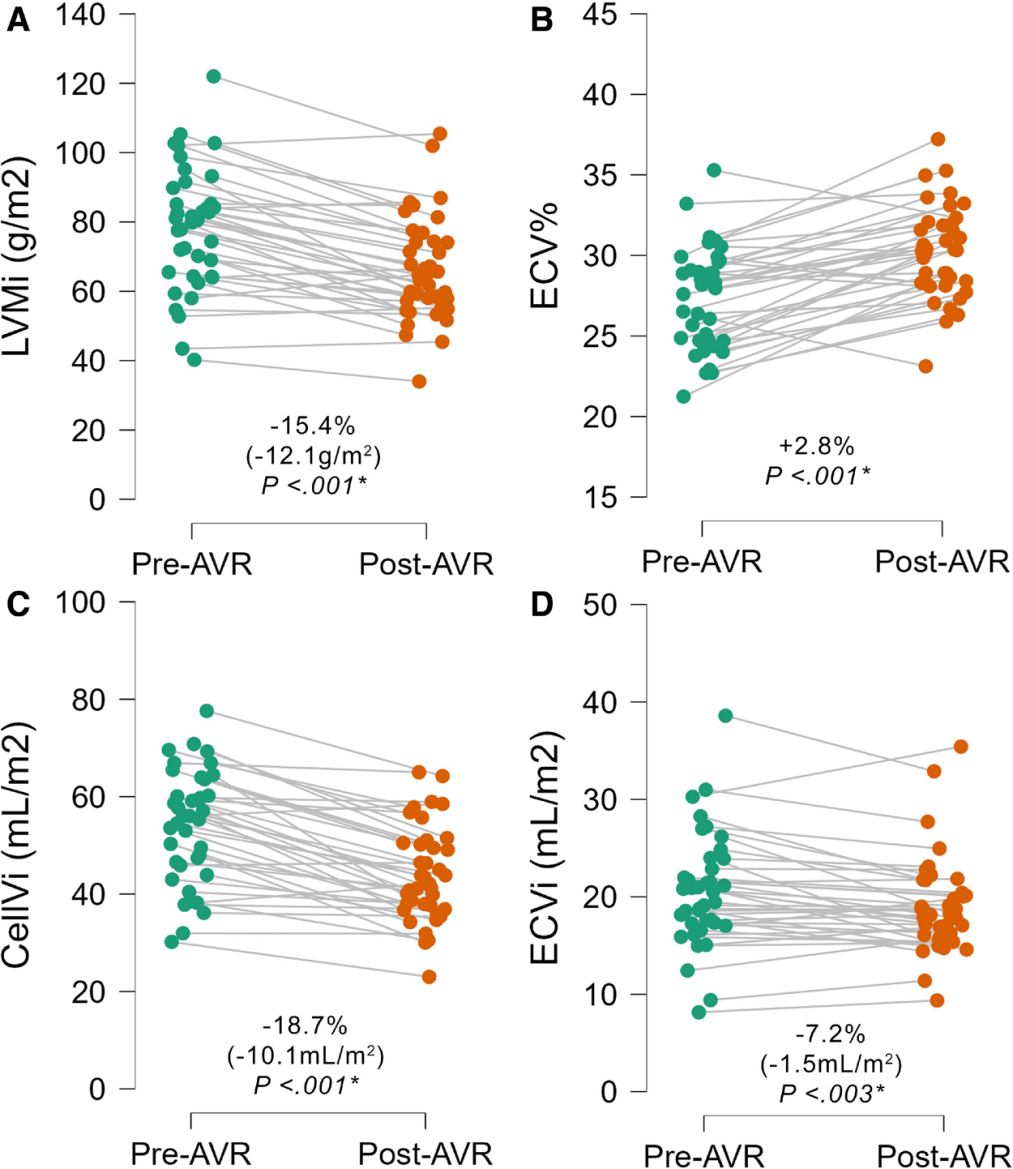
**Raincloud plots comparing pre- and post-aortic valve replacement (AVR) left ventricular remodelling and fibrosis.** There is significant reduction in left ventricular mass index (LVMi; **A**), increase in extracellular volume fraction (ECV%; **B**), and reductions in cellular volume index (CellVi; **C**), and extracellular volume index (ECVi; **D**) post-AVR.

**Figure 3.**
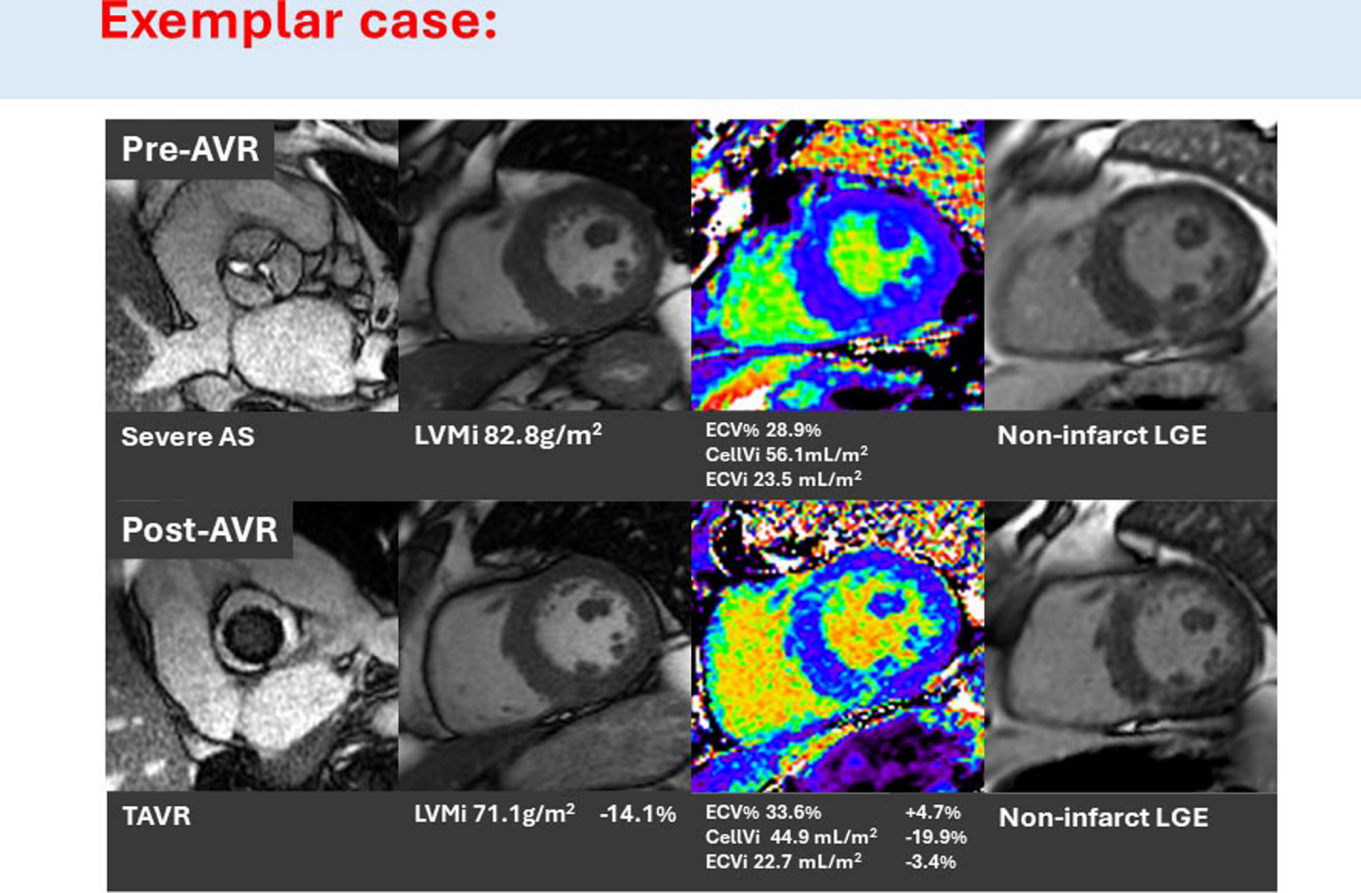
**Exemplar case of changes in left ventricular remodeling and tissue changes pre- and post-aortic valve replacement (AVR) in a patient undergoing transcatheter AVR (TAVR).** AS indicates aortic stenosis; CellVi, cellular volume index; ECV%, extracellular volume fraction; ECVi, extracellular volume index; LGE, late gadolinium enhancement; and LVMi, left ventricular mass index.

At follow-up, there was a significant 15.4% reduction in LVMi (78.3±17.6 to 66.2±14.8 g/m^2^; *P*<0.001*) and 11.4% reduction in maximal wall thickness (15±2 to 13±2 mm; *P*<0.001*; Table 2). There was no significant change in indexed LV end-diastolic volume (68.3±19.5 to 63.5±14.7 mL/m^2^; *P*=0.10), LVEF (78.4±9.9% to 76.5±9.5%; *P*=0.20), or LAVi (42.8±19.9 to 41.3±15.8 mL/m^2^; *P*=0.52; Figures 2 and 3). There was no statistically significant change in GLS pre- and post-AVR (−14.9±3.2% to −15.4±2.7%; *P*=0.34).

Time from intervention to follow-up did not significantly associate with the change in LVMi (β, −0.515; *P*=0.42) or ECV% (β, −0.018; *P*=0.91) when adjusted for pre-AVR LVMi and ECV%, respectively.

Compared with SAVR, there was no significant difference observed in indexed LV end-diastolic volume, LVMi, maximal wall thickness, or GLS. TAVR patients had significantly elevated LVEF at follow-up compared with SAVR patients after adjustment for baseline LVEF (TAVR 80.2±9.7% versus SAVR 73.9±8.6; *P*=0.040). LAVi was significantly elevated in TAVR patients at follow-up (TAVR 48.2±18.1 versus 36.6±12.3 mL/m^2^; *P*=0.022*); however, after adjustment for baseline LAVi, this was no longer significant (*P*=0.33; Table 3).

### Tissue Characterization Changes at Follow-Up

Early post-AVR, there was a significant 18.7% reduction in CellVi (54.0±11.5 to 43.9±9.7 mL/m^2^; *P*<0.001*) and 7.2% reduction in ECVi (20.6±5.8 to 19.1±4.9 mL/m^2^; *P*<0.001*; Figure 2; Table 2). This unbalanced reduction in both CellVi and ECVi resulted in a significant 2.8% increase in ECV% (27.4±3.1% to 30.2±2.8%; *P*<0.001*). There was no significant difference in native T1 (1037±35 to 1028±34 ms; *P*=0.14), nor presence of new LGE at follow-up.

There was no difference in tissue characterization (native T1, ECV%, CellVi, or ECVi) between SAVR and TAVR post-AVR (Table 3).

### Associations With Cell Volume Regression Post-AVR

Post-AVR, a greater reduction in CellVi was univariately associated with increased baseline LVMi (β, −0.20; *P*<0.001*), lower follow-up diastolic blood pressure (β, 0.31; *P*=0.015*), improved (ie, more negative) GLS (β, 0.85; *P*=0.016*), increased baseline CellVi (β, −0.34; *P*<0.001*), and ECVi (β, −0.43; *P*=0.031*; Table 4). Age, sex, and mode of intervention were not significantly associated with CellVi reduction.

**Table 4.**
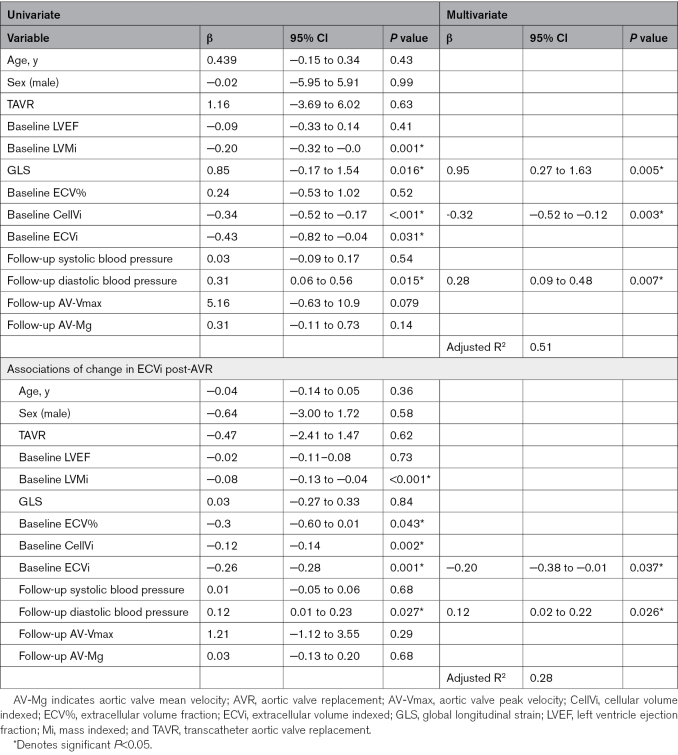
Associations of Change in CellVi Post-AVR

On multivariate analysis, CellVi reduction was independently associated with increased baseline CellVi, improved baseline GLS, and lower follow-up diastolic blood pressure (adjusted-R^2^, 0.51).

### Associations With Extracellular Volume Regression Post-AVR

Change in ECVi was univariately associated with increased baseline LVMi (β, −0.08; *P*<0.001*), CellVi (β, −0.12; *P*=0.002*), ECVi (β, −0.26; *P*<0.001*), ECV% (β, −0.30; *P*=0.043), and lower follow-up diastolic blood pressure (β, 0.12; *P*=0.027; Table 4). On multivariate analysis, ECVi reduction was independently associated with baseline ECVi and follow-up diastolic blood pressure (adjusted-R^2^, 0.28).

### Early Remodeling and Interaction With GLS

**Figure 4.**
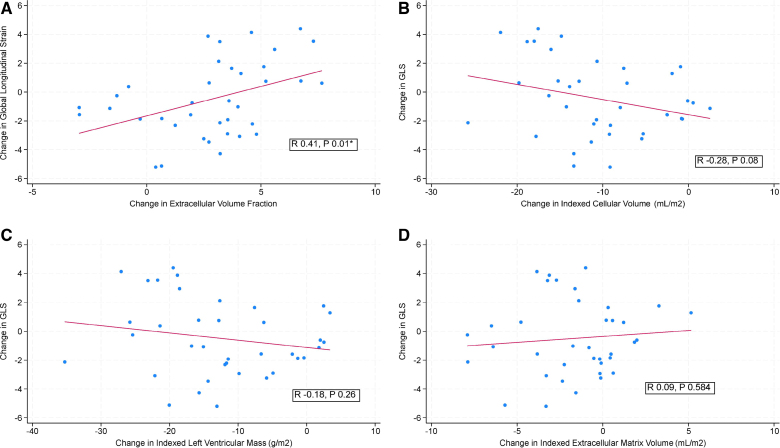
**Scatter graphs for associations pre- and post-aortic valve replacement.** There is significant positive association between change in extracellular volume fraction and global longitudinal strain (GLS; **A**), no significant associations detected for change in cellular volume fraction (**B**), left ventricular mass index (**C**), or extracellular volume index (**D**).

An increase in ECV% early post-AVR was significantly associated with a deterioration in GLS (Pearson r=0.41; *P*=0.01*; Figure 4), while a decrease in CellVi was associated with a deterioration in GLS (Pearson r=−0.28; *P*=0.08), though it did not reach statistical significance. No statistically significant association was observed for change in LVMi (Pearson r=−0.18; *P*=0.26) or change in ECVi (Pearson r=0.09; *P*=0.58).

## Discussion

In this study, we demonstrate for the first time that within 8 weeks of AVR for severe AS, substantial LVH regression occurs with early myocardial plasticity in both cellular and extracellular compartments following afterload relief. Cellular compartment regression is greater than extracellular regression, which leads to an apparent increase in ECV%. Early post-AVR, there was no significant change in GLS despite tissue remodeling. The mode of intervention did not affect the degree of reverse remodeling, indicating that both are effective at resulting in beneficial reverse remodeling post-AVR.

We previously demonstrated a 19% reduction in LVMi at 1 year,^2^ with others showing a 15% LVMi reduction at 6 months.^13,14^ We now show that a significant proportion of the anticipated reduction in LVMi has already occurred by 8 weeks. Indeed, LVMi reduction starts early post-TAVR with a 10% reduction reported within 4 days of the procedure.^15^ Historical histological investigation^16^ suggested that myocardial cell ultrastructural changes are highly reversible post-AVR, and our data add to the body of work confirming that conclusion.

Whereas previous early studies by CMR and echo were limited to overall LVMi, we are now offering insight into the differential regression of its constituents. Utilizing CMR’s superior precision in cardiac chamber assessment and tissue characterization with T1 mapping, our study demonstrates that the early regression in LVH predominantly involves a reduction in the cellular compartment of 18.7%, accounting for a significant amount of the LVMi reduction observed at the 1-year time point.^2^ Notably, our findings also show for the first time that at this early stage dynamic alterations in the extracellular compartment are detectable with a 7.2% reduction also contributing to the early LVH regression. Our findings also highlight that extracellular (diffuse fibrosis) regression appears to be slower than cellular regression (only reaching less than half of the regression expected at 1 year).

Our study presents a discrepancy with a prior CMR study by Flett et al^17^ that did not detect extracellular compartment reduction at 6 months post-AVR but only the cellular compartment reduction at 6 months. However, Flett et al utilized a legacy, now superseded approach to ECV% quantification, namely multibreath-hold T1 quantification equilibrium contrast CMR that suffered from lower precision and greater noise (resulting in greater standard deviations of the results and hence nonsignificant changes at 6 months^17^), which is the likely cause of this discrepancy in findings.

This unbalanced reduction in the cellular and extracellular compartments results in an apparent increase in ECV%, which may explain why we see no improvement in GLS despite the LVMi regression. Additionally, we did not observe further replacement fibrosis, on LGE imaging, at early post-AVR that may have contributed to these findings. Prior research indicates that post-AVR quantified LGE does not increase, however ECV% does.^2,18,19^

Our findings confirm noninvasively by CMR historical observations on histology, where muscle fiber diameter reduced early post-AVR, however interstitial fibrosis had not reduced to the same degree; therefore, there is a relative increase compared with muscular tissue that contributes to diastolic stiffness.^20^ The increased relative interstitial fibrosis concentration entraps muscle and impairs distensibility.^21^ Our finding that an increase in the ECV% early post-AVR is associated with a deterioration in GLS supports the hypothesis that this imbalance between muscle and interstitial fibrosis can additionally impair myocardial function.

Pre-AVR, there were no significant differences observed between the SAVR and TAVR groups regarding LV remodeling, GLS, diffuse reactive fibrosis (ECV%), or focal fibrosis (LGE). As expected, the TAVR group was significantly older and had significantly increased LAVi pre-AVR. Importantly, mode of intervention did not influence the extent of reverse remodeling post-AVR with no impact on degree of LVMi reduction, GLS, or change in tissue characteristics. Therefore, SAVR and TAVR can be considered equally effective at inducing favorable remodeling outcomes. Also, this allows a fairer comparison to prior studies in which the cohorts predominantly consisted of SAVR patients due to the less widespread availability of TAVR.

We also show that those with the highest baseline LVMi had the greatest reductions. A lesser degree of early LVMi reduction has been shown to increase the risk of rehospitalization for heart failure in the initial year following AVR,^6^ and this finding extends long term up to 5 years.^22^ CMR may, therefore, serve as a valuable tool for risk stratification in post-AVR patients shortly after the procedure. This may enable early identification of patients with a lesser degree of LVMi reduction, warranting enhanced monitoring or pharmacological interventions targeting the myocardial remodeling with the aim to mitigate patients increased post-AVR risk. Targeting both cellular and fibrosis regression pharmacologically may therefore be beneficial to reduce post-AVR hospitalization for heart failure. Randomized trials are currently underway to investigate this.^23,24^

Animal study^25^ inducing chronic pressure overload through aortic banding tracked expression of matrix metalloproteinases and their inhibitors before banding, during, and after corrective surgery. Expression increased in response to aortic banding with a return to baseline after corrective surgery. This dynamic regulation of the extracellular matrix likely underpins the noninvasive findings by CMR imaging.

A reduction in CellVi was independently predicted by a higher baseline CellVi, better baseline GLS, and lower follow-up diastolic blood pressure. This supports the evidence highlighting that pre-AVR impaired GLS is adversely prognostic^26–28^ and associated with impaired remodeling post-AVR.^29^ Also, attention should be paid to blood pressure control, as this is a modifiable component that influences both CellVi and ECVi reduction.

An alternative explanation for the observed elevation in ECV% post-AVR may be increased vasodilation at rest, but compensatory vasodilatation tracks cellular demand (so we would assume a reduction in vasodilatation with LVM regression), and in our study, no concomitant increase in native myocardial T1 post-AVR was seen. Also, reduced capillary density in AS^30^ may be less able to accommodate the blood volume changes to exclusively attribute the ECV% increase solely to plasma volume expansion.

We did not detect a significant change in GLS early post-AVR, and to the best of our knowledge, our study is the first to describe changes in GLS at this time point using feature tracking CMR. Previous studies, using both echocardiography and CMR, focused on longer-term follow-up, typically at 1 year, and reported a consistent improvement in GLS at that time point.^31–33^ Studies focusing on early follow-up report more variable results with some detecting regional improvement in strain as early as 3 to 7 days post-TAVR,^26,34^ while Al-Rashid et al^35^ reported no change in GLS at 1 week on echocardiography but improvements at 3 months. This indicates that post-AVR contractility has yet to improve in our cohort, and the benefits associated with improvements in GLS^31^ occur after our assessed time point.

The ability of CMR to evaluate cellular and extracellular compartment regression post-AVR, alongside an improved knowledge of the timeline of this process, may open new therapeutic pathways for improving long-term outcomes. There is an emerging focus on therapeutic agents that target cardiac fibrosis^36,37^ with AS being a suitable condition to potentially trial these agents due to the high degree of fibrosis exhibited and the dynamic nature of it post-AVR. Our findings suggest that targeting fibrosis early after AVR may be beneficial, as unbalanced regression in the early post-AVR period, characterized by an increase in ECV%, is associated with deterioration in GLS in some patients. Cellular hypertrophy is highly plastic and regresses early with successful intervention. Instead, targeting fibrosis with pharmacotherapies (angiotensin-converting enzyme inhibitors, mineralocorticoid receptor antagonists, and sodium-glucose cotransporter 2 inhibitors) may result in balanced reduction in both compartments, earlier GLS improvement, and ultimately improved outcomes.

### Limitations

In this study, we applied fully automated machine learning algorithms for analysis^8,9^ and highly reproducible precision CMR analysis tools,^18^ which have allowed the detection of reverse remodeling and tissue characterization with a comparatively small cohort. Our patients report a significant improvement in functional status post-AVR; however, with a limited follow-up period, this has not been linked to a long-term outcome.

Our method of ECV% quantification, while excluding areas of LGE with infarct, does include nonischaemic LGE that contributes to the overall value. This method is in line with guidelines^38^ and prior investigation,^2^ demonstrating that LGE does not change post-AVR and that the mechanisms involved in diffuse reactive fibrosis and focal replacement fibrosis differ.

Also, our cohort is predominantly male (79%); therefore, assessing sex differences in remodeling post-AVR in AS was limited. This could be a further avenue of investigation as prior research^39,40^ has demonstrated sex differences in collagen and matrix metalloproteinase activity that associated with differing pattern of remodeling pre- and post-AVR. However, the specific changes in the cellular and extracellular components are underexplored.

### Conclusions

Within 8 weeks following AVR for severe AS, a considerable proportion of the reverse remodeling observed at 1 year has already occurred. The regression of LV mass can be attributed not only to the expected decrease in the cellular compartment but also to a reduction in the extracellular compartment, attributable to diffuse reactive fibrosis. This demonstrates the early adaptability of both cellular and matrix compartments in response to relief of the afterload exerted by the stenotic valve. The cellular compartment exhibits a greater degree of plasticity compared with the extracellular compartment at this early stage and may therefore be a target for pharmacological intervention to accelerate fibrosis regression and restore the balance of muscle and fibrosis in the myocardium.

## Article Information

### Acknowledgments

The authors acknowledge the contributions of individuals who made this research possible. Dr Bennett contributed significantly to the statistical analysis and paper preparation. Drs Thornton, Aziminia, Nitsche, Gama, Gul, Shetye, and Bennett contributed in patient recruitment, imaging analysis, manuscript review. Drs Kellman, Davies, Moon, and Treibel provided scientific input, oversight, and review of manuscript. The authors acknowledge the Barts Cardiothoracic and Imaging Group for ongoing support of integrating the clinical and research environments at their local institution.

### Sources of Funding

This study was in part supported by the British Heart Foundation (Dr Thornton [FS/CRTF/21/24128], Dr Treibel [FS/19/35/34374]). Drs Moon, and Treibel are directly or indirectly supported by the University College London Hospital and Barts National Institute for Health and Care Research Biomedical Research Centres and through a British Heart Foundation Accelerator Award. Drs Bennett and Treibel are supported by the European Commission through a Horizon 2020 grant [848109].

### Disclosures

None.

### Supplemental Material

Table S1

## Supplementary Material



## Appendix

Barts Cardiothoracic and Imaging Group (Alphabetical)

Benjamin Adams^5^, Shirish Ambekar^5^, Dincer Aktuerk^5^, Wael Awad^5^, Andreas Baumbach^2,6^, Sanjeev Bhattacharyya^2,6^, Carmelo Di Salvo^5^, Shyamsunder Kolvekar^5^, Kulvinder Lall^5^, David Lawrence^5^, Miss Ana Lopez-Marco^5,6^, Guy Lloyd^2,6^, Simon Kennon^2^, Charlotte Manisty^1,2^, Anthony Mathur^2,6^, Rok Mravljak^2^, Michael Mullen^2^, Aung Oo^5,6^, Mick Ozkor^2^, Iain Pierce^1,2^, Neil Roberts^5^, Amir Sheikh^5^, Alex Shipolini^5^, Rakesh Uppal^5,6^, Phooi Kit Wong^5^, Martin Yates^5^, John Yap^5^

1. Institute of Cardiovascular Science, University College London, London, United Kingdom
2. Cardiovascular Imaging Department, Barts Heart Centre, London, United Kingdom
3. Division of Cardiology, Medical University of Vienna, Vienna, Austria
4. National Heart, Lung, and Blood Institute, National Institutes of Health, Bethesda, MD
5. Department of Cardiothoracic Surgery, Barts Heart Centre, London, United Kingdom
6. William Harvey Research Institute, Queen Mary University, London, United Kingdom
