# Supplementary material for: Left Ventricular Hypertrophy in Aortic Stenosis: Early Cell and Matrix Regression 2 Months Post-Aortic Valve Replacement
Source: Circ Cardiovasc Imaging. 2024 Dec 4;17(12):e017425. doi: 10.1161/CIRCIMAGING.124.017425 (PMC11649182; doi:10.1161/CIRCIMAGING.124.017425)
Supplement: Supplementary file 1 [file hci-17-e017425-s001.pdf]

SUPPLEMENTAL MATERIAL

Table S1:			
SAVR vs TAVR at pre-AVR			
	SAVR:	TAVR:	P-value:
Age, years	66.9 ±8.1	77.8 ±8.6	<.001*
Sex, male (%)	19 (82)	12 (75)	0.69
Diabetes (%):	3 (13)	5 (31)	0.23
Hypertension (%):	16 (69)	10 (62)	0.73
Hypercholesterolaemia (%):	12 (52)	6 (38)	0.51
Atrial fibrillation (%):	2 (22)	3 (19)	0.79
Coronary artery disease (%):	5 (21)	3 (19)	0.82
Systolic/diastolic blood pressure (mmHg):	130 ±16<br>73 ±11	151 ±23<br>80 ±14	0.002*<br>0.10
Echocardiography:			
AV-Vmax, m/s:	4.4 ±0.5	4.3 ±0.5	0.45
LV remodeling:			
LVEDVi, mL/m^2:	68.4 ±18.5	68.1 ±21.6	0.96
LVEF, %:	77.5 ±8.9	79.6 ±11.5	0.53
LVMi, g/m^2:	78.5 ±20.3	78.0 ±13.3	0.94
MWT, mm:	15 ±2	15 ±2	0.94
LAVi, mL/m^2:	35.7 ±15.7	51.1 ±21.6	0.021*
GLS, %:	-15.3 ±3.2	-14.3 ±3.4	0.36
Tissue characterisation:			
Native T1, ms:	1038 ±39	1035 ±28	0.79

ECV%, %:	27.3 ±3.7	27.4 ±2.2	0.88
Infarct LGE, %:	10 (43)	3 (19)	0.16
Non-infarct LGE, %:	17 (74)	12 (75)	1.00
AV-Vmax – aortic valve maximum velocity, AV-Mg – aortic valve mean gradient, LV – left ventricle, LVEDVi – indexed left ventricular end diastolic volume, LVEF – left ventricular ejection fraction, LVMi – indexed left ventricular mass, MWT – maximal wall thickness, LAVi – indexed left atrial volume, ECV% - extracellular volume fraction, LGE – late gadolinium enhancement, GLS – Global longitudinal strain			
** denotes significant p <0.05*			
